# Quality assessment of DNA derived from up to 30 years old formalin fixed paraffin embedded (FFPE) tissue for PCR-based methylation analysis using SMART-MSP and MS-HRM

**DOI:** 10.1186/1471-2407-9-453

**Published:** 2009-12-21

**Authors:** Lasse S Kristensen, Tomasz K Wojdacz, Britta B Thestrup, Carsten Wiuf, Henrik Hager, Lise Lotte Hansen

**Affiliations:** 1 Institute of Human Genetics, University of Aarhus, The Bartholin Building, Wilhelm Meyers Allé 4, DK-8000 Aarhus C, Denmark; 2 Bioinformatics Research Center (BiRC), C. F. Møllers Alle 8, Building 1110, Aarhus University, 8000 Århus C, Denmark; 3 Department of Pathology Aarhus University Hospital Norrebrogade 45, DK-8000 Aarhus C, Denmark

## Abstract

**Background:**

The High Resolution Melting (HRM) technology has recently been introduced as a rapid and robust analysis tool for the detection of DNA methylation. The methylation status of multiple tumor suppressor genes may serve as biomarkers for early cancer diagnostics, for prediction of prognosis and for prediction of response to treatment. Therefore, it is important that methodologies for detection of DNA methylation continue to evolve. Sensitive Melting Analysis after Real Time - Methylation Specific PCR (SMART-MSP) and Methylation Sensitive - High Resolution Melting (MS-HRM) are two methods for single locus DNA methylation detection based on HRM.

**Methods:**

Here, we have assessed the quality of DNA extracted from up to 30 years old Formalin Fixed Paraffin Embedded (FFPE) tissue for DNA methylation analysis using SMART-MSP and MS-HRM. The quality assessment was performed on DNA extracted from 54 Non-Small Cell Lung Cancer (NSCLC) samples derived from FFPE tissue, collected over 30 years and grouped into five years intervals. For each sample, the methylation levels of the *CDKN2A *(*p16*) and *RARB *promoters were estimated using SMART-MSP and MS-HRM assays designed to assess the methylation status of the same CpG positions. This allowed for a direct comparison of the methylation levels estimated by the two methods for each sample.

**Results:**

*CDKN2A *promoter methylation levels were successfully determined by SMART-MSP and MS-HRM in all 54 samples. Identical methylation estimates were obtained by the two methods in 46 of the samples. The methylation levels of the *RARB *promoter were successfully determined by SMART-MSP in all samples. When using MS-HRM to assess *RARB *methylation five samples failed to amplify and 15 samples showed a melting profile characteristic for heterogeneous methylation. Twenty-seven of the remaining 34 samples, for which the methylation level could be estimated, gave the same result as observed when using SMART-MSP.

**Conclusion:**

MS-HRM and SMART-MSP can be successfully used for single locus methylation studies using DNA derived from up to 30 years old FFPE tissue. Furthermore, it can be expected that MS-HRM and SMART-MSP will provide similar methylation estimates when assays are designed to analyze the same CpG positions.

## Background

More than two decades of research have identified DNA methylation of the carbon-5 position of cytosine residues followed by guanine (CpG dinucleotides) as a very important epigenetic mechanism involved in the development of human cancer. Numerous tumor suppressor genes undergo *de novo *methylation and silencing in various cancers despite an overall reduced methylation content of the cancer genome. These changes have shown to be widely implicated in the development and progression of human cancers [1]. Furthermore, the aberrant methylation events take place early in tumorigenesis, and may therefore be used as a potential biomarker for early cancer detection [2].

A sufficient number of clinical samples of high DNA quality are often unavailable for retrospective studies involving DNA methylation biomarkers and studies of other clinically important questions relating to DNA methylation. On the other hand, DNA samples from Formalin Fixed Paraffin Embedded (FFPE) tissue are often abundant and have been stored for decades. However, the use of DNA from old FFPE tissue may be problematic as the DNA is often degraded and only limited amounts may be available. Furthermore, sodium bisulfite treatment [3], preserving methylation marks, and as such necessary for PCR-based studies of DNA methylation, may further degrade the DNA.

Many different PCR-based methods for the detection of DNA methylation have been developed [4]. Methylation specific PCR (MSP) [5] is the most widely used as it is very sensitive, cost-effective, and does not require specialized equipment. However, MSP has several drawbacks. First, it is a non-quantitative method. Measurement of the methylation level in a sample may be relevant, since low-level methylation may not be associated with transcriptional silencing [6]. Second, MSP is prone to false-positive results [7-9]. Third, MSP is not a closed-tube method, which increases the risk of PCR contamination. In a technique called MethyLight [10], the use of TaqMan probes located in between the MSP primers allow for methylation levels to be estimated. This, however, increases the costs of the experiments.

The second range of PCR-based methods, used in locus specific methylation studies, amplifies the DNA template regardless of its methylation status, which can be analyzed post-PCR by a variety of different platforms [3,11-14]. However, it is often difficult to achieve a methylation-independent PCR amplification due to the PCR bias phenomenon, which is described as preferential amplification of unmethylated templates [15,16].

In collaboration with A. Dobrovic and colleagues, we have recently developed two methods for the analysis of DNA methylation aiming at overcoming some of the problems of previously described methodologies. Both methods utilize the High-Resolution Melting (HRM) technology [17]. The first, Methylation-Sensitive HRM (MS-HRM) [18,19], relies on primers designed to amplify the target independent of its methylation status. The second, Sensitive Melting Analysis after Real Time - Methylation Specific PCR (SMART-MSP) [7], utilizes primers designed to amplify only methylated templates as is the case for MSP and MethyLight. SMART-MSP allows quantifying methylation levels in a cost-effective manner, by using intercalation dyes instead of fluorescent probes. Furthermore, SMART-MSP allows some false-positive results to be excluded from the experiments via HRM analysis, which is an integral part of the protocol [7]. Both methods have shown to be highly sensitive, and allow estimation of the methylation levels in the samples [7,18]. Estimation of methylation levels in MS-HRM is performed on the basis of a comparison of melting profiles of screened samples and standards of known ratios of methylated and unmethylated DNA, whereas in SMART-MSP the real-time PCR data are used to quantify methylation levels. The main feature of MS-HRM, as described by Wojdacz and Dobrovic [20], is that it allows controlling of the PCR bias during methylation-independent PCR amplification. This is achieved by including a limited number of CpG sites in the primers and optimizing the annealing temperature [16,20]. The same real-time PCR and HRM instrument and reagents are used for both methods facilitating independent result verifications.

We have assessed the quality of DNA extracted from archival Non-Small Cell Lung Cancer (NSCLC) FFPE tissue for DNA methylation analysis using the SMART-MSP and MS-HRM methodologies. SMART-MSP and MS-HRM assays were designed to analyze the same CpG positions to allow a direct comparison of the methylation level of the samples, dating 5 - 30 years back in time. The methylation status of the *CDKN2A *(*p16*) and *RARB *promoter CpG islands were analyzed. Variation amongst replicates and amongst bisulfite modifications was evaluated. Furthermore, immunohistochemical staining for the p16 protein was performed and correlated to the methylation status of the samples.

## Methods

### Samples and DNA Extraction

FFPE blocks from 54 Non-Small Cell Lung Cancer (NSCLC) patients were selected from the archives of the Institute of Pathology, Aarhus University Hospital. Seven samples were 30 years old, eight samples were 25 years old, 13 samples were 20 years old, 10 samples were 15 years old, 10 samples were 10 years old, and six samples were five years old. Blocks containing large areas of carcinoma infiltration were selected. For each sample, four tissue sections of 10 μm were transferred into a microcentrifuge tube and incubated in 1.5 ml of xylene for 30 min at 37°C. After centrifugation at 14,000 rpm for 3 min, the supernatant was removed. This step was repeated once. Subsequently, the tissue samples were washed in 1 ml of 99% ethanol. After centrifugation at 7200 rpm for 3 min the supernatant was discharged. The washing procedure was repeated twice. The samples were air dried at ambient temperature for 30 min. DNA was extracted using the MagNA Pure LC (Roche Molecular Biochemicals, Penzberg/Germany), a semi-automatic system for isolation of nucleic acids utilizing magnetic bead technology. The purification was done according to the manufacture's instructions.

DNA was extracted from peripheral blood (PB) obtained from medical students of both sexes in their first year at Medical school, following a modified salt precipitation protocol as previously described [21].

The Local Ethical Committee, Aarhus County, Denmark, approved this study.

### Stereological analysis

In order to estimate the amount of tumor tissue and necrosis in the sections and to detect if low level of methylation were due to low amount of vital tumor, we chose to determine the area fraction (AF) of these two components. The stereological analysis was performed using a light microscope equipped with a computer assisted stereology system (CAST, Olympus, Denmark). The counting frame covered an area of 8,320 μm and the sampling was done in a systematic, random fashion. A median number of 18 (range 5-26) and 20 (range 4-40) counting frames were evaluated for vital tumor and necrosis, respectively. The AF staining positive for vital tumor and necrosis was determined by point-counting [22] at a magnification of × 739. Points hitting artifacts were not considered.

### Preparation of control samples

Universal methylated DNA (Chemicon, Millipore, Billerica, MA) was subjected to bisulfite modification as described below and used as a methylation positive control for the SMART-MSP and MS-HRM assays. DNA extracted from PB was subjected to whole genome amplification (WGA) for creation of an unmethylated control. The WGA was performed as previously described [7], in which the primary WGA product was subjected to a second round of WGA. The secondary WGA product was subjected to bisulfite modification, as described below, and used as an unmethylated control. A standard dilution series, of 100%, 10%, 1%, and 0.1% methylated DNA in a background of unmethylated DNA, was prepared by serially diluting the methylation positive control into the unmethylated control. These standards were used for assessing the sensitivity of the SMART-MSP and MS-HRM assays, and for melting profile comparisons with the samples in MS-HRM. For the SMART-MSP assays the standard dilution series were, in addition, used to assess the quantitative accuracy and the PCR efficiency of the assays using the LightCycler 480 Software Version 1.5.

### Sodium Bisulfite Treatment

Five hundred nanograms of genomic DNA was subjected to sodium bisulfite treatment with the EpiTect^® ^Bisulfite kit (Qiagen, Hilden, Germany) according to the manufacturer's instructions.

### MS-HRM and SMART-MSP primer design

**Figure 1:**
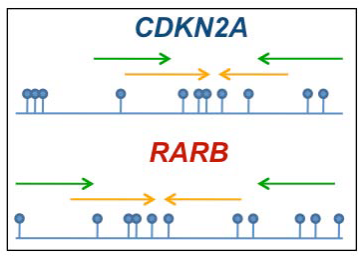
**The design of the *CDKN2A *and *RARB *SMART-MSP and MS-HRM assays**. The SMART-MSP assays were designed to analyze the same CpG positions as the MS-HRM assays to allow for a direct comparison of the methylation levels in the samples. The MS-HRM primers are denoted as green arrows and the SMART-MSP primers as orange arrows. The CpG positions are denoted as lollipops.

MS-HRM primers were designed to be methylation independent according to the guidelines given in [20], and the SMART-MSP primers were designed to be methylation specific according to the guidelines given in [7]. The SMART-MSP assays were designed to assess the same CpG positions as the MS-HRM assays (Figure 1) to allow for a direct comparison of the methylation levels estimated by the two methods. For this reason, it was not possible to include CpG sites between the SMART-MSP primers to allow for the detection of false positive results due to false priming events as described [7]. Both SMART-MSP assays have one non-CpG cytosine between the primers. The conversion status of these cytosines can be assessed by the HRM analysis, and a right-shifted melting profile indicate that the observed amplification may be due to incomplete conversion of unmethylated cytosines [7]. The primer sequences and genomic regions spanned, as well as amplicon sizes and the annealing temperatures (T_A_) are listed in Table 1.

**Table 1:** Primer sequences, annealing temperatures, and amplicon information for the MS-HRM and SMART-MSP assays (UCSC Genome Browser, August 2008).

Gene	Primer sequences (CpG sites underlined and converted Cs as capital Ts or As). (5'→ 3').	Annealing temperature	Amplicon size	CpGs/non-CpG-Cs between the primers	**Spanned region(bp)**
*CDKN2A* (MS-HRM)	F-ggagTTttcggTtgaTtggTtggTT R-aAcaAcgcccgcacctcctcta	64°C	69 bp	5/3	21964733-21964800 of Chr. 9
*RARB* (MS-HRM)	F-cgagTtgtttgaggaTtgggatgT R-aatAcgttccgAatcctacccc	64°C	89 bp	7/5	25444840-25444928 of Chr. 3
*CDKN2A* (SMART-MSP)	F-gTtgaTtggTtggTTacgTcgc R-ctcctctacccgaccccgA	64°C	45 bp	0/1	21964746-21964790 of Chr. 9
*RARB* (SMART-MSP)	F-gggatgTcgagaacgcgagc R-cgAtAcccaAacaaaccctActcg	64°C	48 bp	0/1	25444857-25444904 of Chr. 3
ALU control (SMART-MSP)	F-ggttaggtatagtggtttatatttgtaattttagta R-attaactaaactaatcttaaactcctaacctca	62°C	98 bp	N/A	Consensus seq., See ref [23].

### SMART MSP control assay

In SMART-MSP a control assay was used to normalize for DNA input in the reactions as DNA concentrations cannot be measured directly after bisulfite modification. We have used a control assay based on Alu sequences depleted of CpG sites by evolutionary deamination previously described [23]. This assay is less susceptible to normalization errors caused by aneuploidy and copy number changes often observed in cancer cells, and is especially suitable for determining relative DNA amounts in samples where the quantity and/or quality may be limited [23]. Here, this assay was successfully applied for SMART-MSP quantification without the TaqMan probe using an intercalating fluorescent dye instead.

#### PCR and HRM Conditions

PCR cycling and HRM analysis were performed on the LightCycler^® ^480 (Roche Applied Science, Mannheim, Germany). The reaction mixtures consisted of 20 ng of bisulfite modified DNA using the LC480 HRM Scanning Master (Roche) with primer concentrations of 200 nmol/l of each primer and a final MgCl_2 _concentration of 3 mmol/l in a total volume of 10 μl. The cycling protocol started with one cycle of 95°C for 10 minutes for enzyme activation followed by 45 cycles of 95°C for 5 seconds, T_A _(Table 1) for 10 seconds, 72°C for 10 seconds, one cycle of 95°C for 1 minute, one cycle of 65°C for 1 minute and a melt from 65 to 95°C for all assays. In the ALU control assay the PCR cycling steps was performed for 10, 20, 20 seconds instead of 5, 10, 10 seconds. The melting step was performed using 30 acquisitions per °C. For the MS-HRM assays the annealing temperatures were experimentally determined for each assay to ensure that the 0.1% methylated standard could be detected. For the SMART-MSP assays the annealing temperatures were experimentally determined for each assay to ensure that only methylated templates were amplified. All samples were analyzed in triplicate.

### MS-HRM and SMART-MSP data analysis

In MS-HRM, the methylation levels of each sample were assessed by comparison of the PCR product melting profiles between each sample and the standards with known ratio of methylated and unmethylated templates as described [18]. Here, the melting profiles of samples were compared to melting profiles of PCR products derived from mixes of 100%, 10%, 1%, 0.1% and 0% of fully methylated template in an unmethylated background, and scored as being methylated at low level (0.1-1%) medium level (1-10%) or high level (10-100%). The samples were scored as being heterogeneously methylated if a melting profile characteristic for the amplification of molecules of different methylation content was observed as previously described [13,24].

In SMART-MSP, the relative methylation levels in the samples were estimated relative to universal methylated DNA (Chemicon) using the 2^-ΔΔCt ^real-time PCR quantification approach ()[25], as described in [7] in which ΔΔCt = unknown sample (Ct_target gene _- Ct_ALU control_) - 100% methylated DNA (Ct_target gene _- Ct_ALU control_). This approach implies that the methylation assays have approximately the same *E *as the control assay ()[25]. The HRM profiles of the samples were compared to the melting profile of universal methylated DNA (Chemicon) and results were discarded if a deviation of more than 0.5ΔC was observed. The melting temperature of each assay was approximately 80.3°C for *CDKN2A *and 77.4°C for *RARB*.

The samples were analyzed in triplicates in order to assess if the reproducibility amongst replicates of the SMART-MSP assays decreased with the age of the samples. For this purpose, three different methylation estimates were calculated from the individual Ct-values of the triplicates of each sample in the control assay and the respective methylation assay. The first value is the lowest possible methylation estimate that can be calculated from the Ct-values (using the highest Ct-value of the methylation assay and the lowest Ct-value of the control assay of the triplicates). The second value is calculated from the average Ct-value of the triplicates, whereas the third value is the highest possible value that can be calculated from the Ct-values (using the lowest Ct-value of the methylation assay and the highest Ct-value of the control assay).

In MS-HRM, at least two of the three replicates of a sample need to give the same result (methylation positive or methylation negative) for the methylation level to be determined. For both methods, samples were scored as "failed amplification" when only one or none of the three replicates amplified.

### The sensitivity and quantitative accuracy of the assays

**Figure 2:**
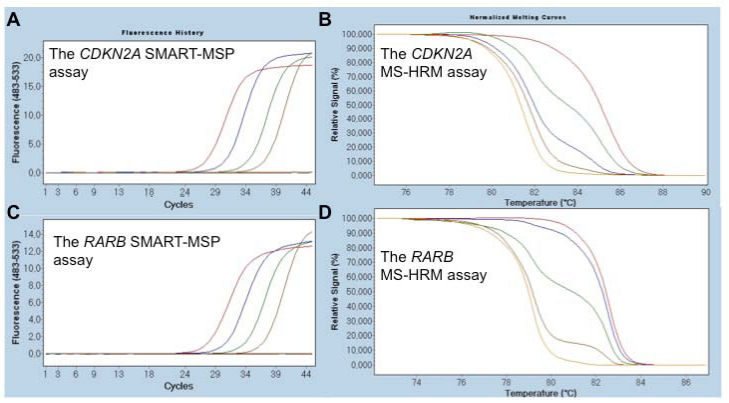
**The sensitivity of the *CDKN2A *and *RARB *SMART-MSP and MS-HRM assays**. The sensitivity of the assays was tested using standard dilution series of methylated DNA into unmethylated DNA. The 100% methylated standards are indicated in red, the 10% methylated standards in blue, the 1% methylated standards in green, the 0.1% methylated standards in brown, the 0% methylated standard in orange and non template controls are indicated in black. **A**. The *CDKN2A *SMART-MSP assay (real-time amplification data). **B**. The *CDKN2A *MS-HRM assay (normalized melting curves). **C**. The *RARB *SMART-MSP assay (real-time amplification data). **D**. The *RARB *MS-HRM assay (normalized melting curves). All assays successfully detect 0.1% methylation.

A standard dilution series from 100% to 0.1% methylated DNA was used to assess the sensitivity of the *CDKN2A *and *RARB *SMART-MSP and MS-HRM assays (Figure 2).

In addition, the standard dilution series was used for the SMART-MSP assays to assess the quantitative accuracy and the PCR efficiency of each assay.

Based on the standard curve the LC480 software measures an *Error *value (mean squared error of the single data points fit to the regression line). The *Error *value is a measurement of the quantitative accuracy of the assays. An acceptable *Error *value should be less than 0.2 according to the LightCycler 480 Instrument Operator's Manual. The *Error *value for the *CDKN2A *assay was 0.13, the *Error *value for the *RARB *assay was 0.10, and the *Error *value for the ALU control assay was 0.13.

The LC480 software was also used to calculate the PCR efficiency (*E*) from the standard curve for each of the SMART-MSP assays. The *E *value for each assay was: *CDKN2A *= 2.01, *RARB *= 2.07, and ALU Control = 2.02.

### Immunohistochemistry

Sections of fixed paraffin-embedded tissue were dewaxed in xylene and rehydrated in graded alcohol. Endogenous peroxidase activity was blocked by 0.5 per cent H_2_O_2 _in absolute methanol for 10 min at room temperature. To reveal antigen, the sections were pretreated by boiling in target reveal solution (DAKO, Denmark) using a microwave oven. Non-specific binding of immunoglobulin was quenched by incubating the sections in 10 per cent goat serum for 20 min. Sections were incubated with a 1:50 diluted mouse-antihuman monoclonal p16 antibody (clone G175-405, monoclonal mouse IgG; Pharmingen, San Diego, CA). Antigen - antibody reaction was detected by incubation with EnVisionTM+ Dual Link System-HRP (DAKO, Denmark). The peroxidase activity was visualized by 0.05 per cent 3,3-diaminobenzidine tetrahydro-chloride (Kem-En-Tek, Denmark) dissolved in PBS containing 0.1 percent H_2_O_2_. Counterstaining was carried out using Mayer's haematoxylin. Finally, the sections were dehydrated in graded alcohol and mounted in hydrophobic mounting media. Negative controls were obtained after incubation of the sections in PBS instead of primary antibodies. Reactive lymphocytes and inflammatory cells served as positive internal controls. A tumor was considered positive if 5% of the nuclei showed nuclear staining.

## Results

### Methylation analysis of the FFPE samples

The methylation levels of the *CDKN2A *and *RARB *promoters in 54 FFPE NSCLC samples, stored between 1978 and 2003, were estimated by the MS-HRM and SMART-MSP assays. The estimates of the methylation levels are given in Table 2. As described in the methods section three different methylation estimates were calculated for each sample when using SMART-MSP to evaluate the variation between replicates. The integrity of the DNA was sufficient for all samples showing 0% methylation in the SMART-MSP assays, as all of these samples amplified in the ALU control assay.

**Table 2:** Methylation levels of the samples as estimated by MS-HRM and SMART-MSP.

		Methylation levels estimated for *CDKN2A*				Methylation levels estimated for *RARB*			
		
Sample	Immunohistochemistry for p16	MS-HRM	SMART-MSP			MS-HRM	SMART-MSP		
					
			Lowest estimate	Average estimate	Highest estimate		Lowest estimate	Average estimate	Highest estimate
**1 (1978)**	Negative	0.1%-1%	1.7%	2.7%	3.4%	Failed ampl	0%	0%	0%
**2 (1978)**	Negative	0%	0% 0%	0% 0%	0% 0%	0.1%-1%	0%	0%	0%
**3 (1978)**	Positive	0%	0% 0%	0% 0%	0% 0%	Het met	0%	0%	0%
**4 (1978)**	Negative	0%	0% 0%	0% 0%	0% 0%	1%-10%	2.4%	9.5%	30.8%
**5 (1978)**	Positive	0%	0% 0%	0% 0%	0% 0%	10%-100%	14.4%	20.3%	28.7%
**6 (1978)**	Negative	0%	0% 0%	0% 0%	0% 0%	0.1%-1%	0.1%	0.3%	0.4%
**7 (1978)**	Negative	0%	0% 0%	0% 0%	0% 0%	0%	0%	0%	0%
**8 (1978)**	No data	10%-100%	20.3%	25.0%	28.7%	10%-100%	57.4%	61.6%	75.8%
**9 (1983)**	Negative	1%-10%	0.7%	1.9%	5.4%	Failed ampl	4.1%	5.8%	7.2%
**10 (1983)**	Positive	0%	0%	0%	0%	10%-100%	21.8%	26.8%	33.0%
**11 (1983)**	Negative	0.1%-1%	1.7%	2.4%	3.3%	Het met	0%	0%	0%
**12 (1983)**	Positive	0%	0%	0%	0%	1%-10%	18.9%	33.0%	53.6%
**13 (1983)**	Negative	1%-10%	19.0%	27.0%	30.8%	0%	0%	0%	0%
**14 (1983)**	Negative	0.1%-1%	0.8%	2.5%	6.3%	Het met	0.2%	0.4%	1.4%
**15 (1983)**	Negative	0%	0% 0%	0%	0%	0.1%-1%	0.3%	0.9%	5.1%
**16 (1988)**	Negative	0%	0%	0%	0%	Het met	0.4%	0.8%	1.3%
**17 (1988)**	Negative	0%	0%	0%	0%	10%-100%	93.3%	114.9%	186.6%
**18 (1988)**	Negative	0%	0%	0%	0%	Failed ampl	0%	0%	0%
**19 (1988)**	Negative	1%-10%	3.4%	5.8%	10.9%	Het met	0.2%	0.8%	3.8%
**20 (1988)**	Negative	10%-100%	12.5%	14.4%	21.8%	0%	0%	0%	0%
**21 (1988)**	Positive	0%	0%	0%	0%	Het met	0.1%	0.5%	2.4%
**22 (1988)**	Positive	0%	0%	0%	0%	Het met	2.2%	2.7%	3.8%
**23 (1988)**	Negative	10%-100%	18.9%	20.3%	21.7%	0%	0%	0%	0%
**24 (1988)**	Negative	10%-100%	9.5%	14.4%	15.4%	Het met	0.1%	0.2%	0.7%
**25 (1988)**	Negative	10%-100%	6.3%	7.7%	10.9%	0%	0%	0%	0%
**26 (1988)**	Negative	0%	0%	0%	0%	Het met	0.2%	0.3%	0.6%
**27 (1988)**	Negative	10%-100%	12.5%	14.4%	15.4%	Failed ampl	0%	0%	0%
**28 (1988)**	Positive	0%	0%	0%	0%	0%	0%	0%	0%
**29 (1993)**	Negative	1%-10%	3.6%	5.8%	7.7%	0%	0%	0%	0%
**30 (1993)**	Positive	0%	0%	0%	0%	1%-10%	2.9%	8.8%	14.4%
**31 (1993)**	Negative	0%	0%	0%	0%	Het met	0.3%	0.7%	1.4%
**32 (1993)**	Negative	10%-100%	21.8%	26.8%	30.8%	0.1%-1%	0.1%	0.3%	0.7%
**33 (1993)**	Negative	0%	0%	0%	0%	0%	0%	0%	0%
**34 (1993)**	Negative	10%-100%	25.0%	40.6%	61.6%	1%-10%	2.9%	5.4%	8.8%
**35 (1993)**	Positive	0%	0%	0%	0%	Het met	0.3%	0.6%	0.8%
**36 (1993)**	Negative	10%-100%	5.1%	10.9%	17.7%	0.1%-1%	0.2%	0.6%	1.4%
**37 (1993)**	Positive	0%	0%	0%	0%	1%-10%	23.3%	25.0%	28.7%
**38 (1993)**	Positive	0%	0%	0%	0%	0%	0%	0%	0%
**39 (1998)**	Negative	0.1%-1%	0%	0%	0%	0%	2.5%	2.9%	3.4%
**40 (1998)**	Negative	0.1%-1%	0.1%	0.1%	0.2%	Het met	0.6%	1.5%	3.1%
**41 (1998)**	Negative	10%-100%	19.0%	23.3%	28.7%	0%	0%	0%	0%
**42 (1998)**	Positive	0%	0.1%	0.3%	0.7%	Het met	0.2%	0.4%	1.4%
**43 (1998)**	Positive	0%	0%	0%	0%	0%	0%	0%	0%
**44 (1998)**	Positive	0.1%-1%	0.1%	0.2%	0.4%	Het met	2.9%	3.6%	5.4%
**45 (1998)**	Positive	0%	0% 0% 0%	0% 0%	0% 0%	0.1%-1%	0.2%	0.7%	2.9%
**46 (1998)**	Positive	0%	0%	0%	0%	0.1%-1%	0%	0%	0%
**47 (1998)**	Positive	0%	0%	0%	0%	10-100%	21.8%	30.8%	50.0%
**48 (1998)**	Positive	0%	0%	0%	0%	Failed ampl	0%	0%	0%
**49 (2003)**	Positive	0%	0%	0%	0%	0%	0%	0%	0%
**50 (2003)**	Positive	0%	0%	0%	0%	Het met	0.5%	1%	2.1%
**51 (2003)**	No data	0%	0%	0%	0%	0%	0%	0%	0%
**52 (2003)**	Positive	0.1%-1%	0.4%	0.6%	1.0%	1%-10%	2.7%	3.9%	5.1%
**53 (2003)**	Negative	10%-100%	2.7%	6.3%	16.5%	1%-10%	4.1%	10.2%	26.8%
**54 (2003)**	Negative	0%	0%	0%	0%	0%	0%	0%	0%

An average methylation estimate as well as the highest and lowest possible methylation estimates calculated from the Ct-values of the triplicates for the gene and the control is given for SMART-MSP.

For *CDKN2A *we could successfully determine the methylation levels of all 54 samples using both methods. In 46 of the 54 samples the average SMART-MSP methylation estimate (second column within the SMART-MSP column in Table 2) was within the range estimated by MS-HRM. This is unlikely to have occurred by chance (Binomial test, p-value 1.384 × 10^-7^).

**Figure 3:**
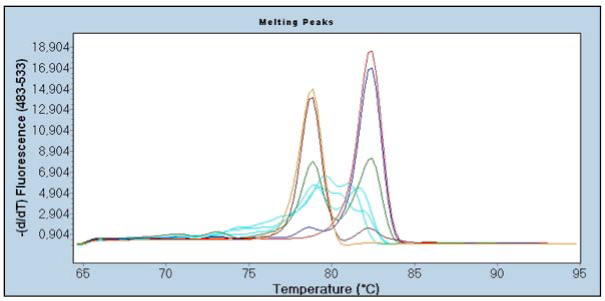
**Melting profiles indicating that the DNA is heterogeneously methylated at the *RARB *promoter**. If molecules, in which different CpG positions are methylated and unmethylated are amplified, the observed melting profile will be complex. These different molecules will form heteroduplexes and homoduplexes after the PCR and prior to the HRM step. The very early melting observed is likely to be the melting of various heteroduplexes. The intermediate and later melting are likely to be the melting of various homoduplexes with those having the highest number of CpG positions methylated melting the latest. The 100% methylated standards are indicated in red, the 10% methylated standards in blue, the 1% methylated standards in green, the 0.1% methylated standards in brown, the 0% methylated standard in orange and non template controls are indicated in black. A sample (in triplicate) judged to be heterogeneously methylated is indicated in turquoise.

Methylation levels for *RARB *were successfully determined when using SMART-MSP of all 54 samples. Five samples failed to amplify when using MS-HRM. The MS-HRM results indicated that heterogeneously methylated molecules were amplified in 15 of the samples. An example of a heterogeneously methylated sample is shown in figure 3. All of the heterogeneously methylated samples were estimated to be methylated at low levels (0%-3.6%) by SMART-MSP. Twenty-seven of the remaining 34 samples had an average SMART-MSP methylation estimate (second column within the SMART-MSP column in Table 2) within the range estimated by MS-HRM. This is unlikely to have occurred by chance (Binomial test, p-value 0.0008214).

**Figure 4:**
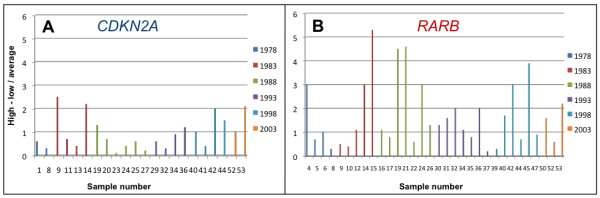
**Reproducibility of the methylation estimates for the FFPE samples by SMART-MSP**. Plots of the absolute difference between the highest and lowest possible methylation estimate divided by the average methylation estimate against the age of the samples for each gene are shown. **A**. The *CDKN2A *assay. **B**. The *RARB *assay. It is observed that the overall reproducibility is better for the *CDKN2A *assay compared to the *RARB *assay. It can also be observed that the reproducibility did not depend on the age of the samples.

To evaluate if the reproducibility of the methylation estimates amongst replicates was dependent on the age of the samples, we plotted the absolute difference between the highest and lowest possible methylation estimate divided by the average methylation estimate against the age of the samples for each gene (Figure 4). From these plots, it was concluded that the reproducibility of the SMART-MSP assays did not decrease with the age of the samples. In general, the reproducibility was better for the *CDKN2A *SMART-MSP assay compared to the *RARB *SMART-MSP assay.

### Percentage of tumor tissue and necrosis versus methylation level

The amount of tumor tissue and necrosis in the sections were determined in order to evaluate if low level of methylation were due to low amount of vital tumor or influenced by the amount of necrosis. However, we found no difference in area of necrosis or vital tumor between the tumors with low, medium or high level of methylation (data not shown). This may be due to the fact that methylation of both *CDKN2A *and *RARB *can be detected in adjacent normal lung tissue [26].

### Immunohistochemistry

**Figure 5:**
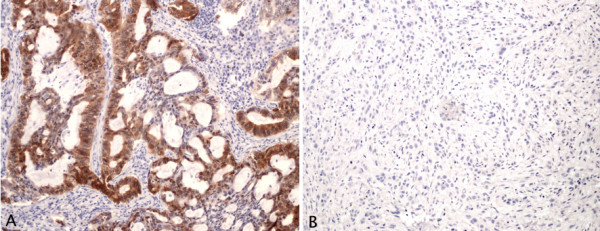
***CDKN2A *(*p16*) expression in NSCLC**. **A**. A case in which immunohistochemical staining for p16 showed positive nuclear and cytoplasmic staining (×100). **B**. A case in which the invading tumor cells did not express p16 (×100).

It is well known that the quality of old FFPE samples is sufficient for immunohistochemistry to be performed. Therefore, we evaluated whether the immunohistochemical staining of the p16 protein correlated with methylation status of the *CDKN2A *(*p16*) gene. An example of a positive and a negative sample is shown in figure 5, and the results can be found in table 2. Immunohistochemistry results were available for all samples except two. Eighteen of the 20 methylated samples for which immunohistochemistry results were available were negative. The two methylated samples that were positive by immunohistochemistry were only found to be methylated at very low levels (0.1%-1% by MS-HRM as well as by SMART-MSP). Of the 30 unmethylated samples for which immunohistochemistry results were available 18 samples were positive. Thus, an association between methylation and immunohistochemical staining was found (Pearson's Chi-squared test (p-value = 0.001192)). Two samples, for which the MS-HRM and SMART-MSP did not give the same methylation status, were omitted from the analysis.

### Variation amongst bisulfite modifications

As the variation among bisulfite modifications previously has been thoroughly evaluated using various PCR-based methods for single locus methylation detection [27,28], we bisulfite modified a subset of the samples a second time, and tested for *CDKN2A *methylation using MS-HRM. Results obtained after the second bisulfite modification were identical to the results obtained by the first bisulfite modification.

## Discussion

DNA derived from FFPE tissue represents a highly valuable source of material for retrospective studies. However, DNA derived from FFPE tissue is often highly degraded, and for certain cancer types a limited amount of tumor tissue may be available. When studying DNA methylation changes by PCR-based methods, it is necessary to modify the DNA with sodium bisulfite for preservation of the DNA methylation information of the original template. This treatment may further damage the DNA. Also, it is likely that the DNA becomes even more fragmented with age of storage.

Here, we have evaluated whether we could use sodium bisulfite treated DNA derived from FFPE tissue from NSCLC patients dating five to 30 years back in time for DNA methylation analysis. Two methodologies, SMART-MSP and MS-HRM, were used to assess the methylation status of each of the 54 samples available for this study. Both methods are highly sensitive and capable of measuring methylation levels.

Assays targeting the promoter CpG islands of the *CDKN2A *and *RARB *genes were designed to evaluate the quality of the samples since aberrant methylation of these genes is implicated in NSCLC [29], and especially *CDKN2A *has shown promising results as a biomarker for early detection [30].

The methylation status of all 54 samples could be determined for both genes using SMART-MSP, even in the oldest samples. When using MS-HRM, all samples could be scored for *CDKN2A *methylation, but five samples could not be scored for *RARB *methylation due to failed amplification in at least two of three replicates. This may be due to the fact that the MS-HRM *RARB *amplicon is almost twice as long as the SMART-MSP *RARB *amplicon (see Table 1). For all amplifiable samples a highly significant correlation was observed between the methylation estimates provided by each method.

One sample was estimated to be methylated above 100% at the *RARB *promoter by SMART-MSP. Methylation estimates relative to *in vitro *methylated DNA may occasionally be obtained for heavily methylated genes because the calibrator (positive control) may not be 100% methylated in spite of extensive SssI treatment [31].

We observed a tendency for the *RARB *promoter to be heterogeneously methylated. It was only possible to examine whether the DNA was heterogeneously methylated, when using MS-HRM as the SMART-MSP assays are designed only to amplify fully methylated molecules. The samples were scored as being heterogeneously methylated, when showing melting profiles characteristic for the amplification of molecules of different methylation content [13,24].

The reproducibility of the SMART-MSP assays could be expected to be less satisfactory for the older samples. To investigate this hypothesis we plotted the absolute difference between the highest and lowest possible methylation estimate for the methylated samples divided by the average methylation estimate against the age of the samples for each gene. From these plots it was clear that the reproducibility did not decrease with the age of the samples (Figure 4). Therefore, we have, for the first time, showed the robustness of SMART-MSP and MS-HRM for methylation analysis of DNA extracted from FFPE tissue being up to 30 years old. The reproducibility was higher for the *CDKN2A *SMART-MSP assay compared to the *RARB *SMART-MSP assay. This may be explained by the tendency for the *RARB *promoter to be heterogeneously methylated in the samples we have tested.

Furthermore, we have evaluated whether immunohistochemical staining of the p16 protein correlated with methylation status of the *CDKN2A *(*p16*) gene. A highly significant correlation was found (p-value = 0.001192), and all methylated samples except two, which were methylated at very low levels, were found to be negative. Twelve of 30 unmethylated samples were also negative by immunohistochemistry. This may be due to homozygous deletion of the *CDKN2A *(*p16*) gene, which often is found in NSCLC [32].

A recent study has assessed the quality of archival FFPE tissue for DNA methylation studies using MS-HRM and MethyLight [27]. A high concordance between the MS-HRM and MethyLight results was observed, and a high reproducibility in between different runs and bisulfite modifications was found. However, no information of the age of the FFPE tissue was provided.

The methylation assays we have developed analyze the same CpG positions in two different ways, using the same real-time PCR and HRM instrument. In spite of the innate differences of the two types of assays, we have shown that similar methylation estimates can be obtained. For this reason, a more accurate and reliable methylation estimate may be obtained when using both SMART-MSP and MS-HRM.

## Conclusions

In conclusion, DNA from FFPE tissue can be successfully used for methylation studies in spite of being 30 years old when using MS-HRM or SMART-MSP. These two methodologies are closed-tube, very sensitive, and require no fluorescent probes. Since MS-HRM and SMART-MSP can be performed on the same instrument, a more accurate methylation estimate can be achieved in the samples of interest, as each method verifies the results of the other.

## Competing interests

The authors declare that they have no competing interests.

## Authors' contributions

Conceived and designed the experiments: LSK, TKW, HH, and LLH. Performed the experiments: LSK, HH and BBT. Analyzed the data: LSK, TKW, BBT, CW, HH, and LLH. Contributed reagents/materials/analysis tools: LLH and HH. Wrote the paper: LSK, TKW, HH, and LLH. All authors have red and approved the final manuscript.

## Pre-publication history

The pre-publication history for this paper can be accessed here:

http://www.biomedcentral.com/1471-2407/9/453/prepub
